# Photocatalytic setup for *in situ* and *operando* ambient-pressure X-ray photoelectron spectroscopy at MAX IV Laboratory

**DOI:** 10.1107/S1600577523002801

**Published:** 2023-04-17

**Authors:** Alexander Klyushin, Manoj Ghosalya, Esko Kokkonen, Calley Eads, Rosemary Jones, Naresh Nalajala, Chinnakonda S. Gopinath, Samuli Urpelainen

**Affiliations:** a MAX IV Laboratory, Lund University, Box 118, Lund 22100, Sweden; b Nano and Molecular Systems Research Unit, University of Oulu, Oulu 90014, Finland; c Division of Synchrotron Radiation Research, Department of Physics, Lund University, Box 118, Lund 22100, Sweden; d Catalysis and Inorganic Chemistry Division, CSIR-National Chemical Laboratory, Pune 411008, India; Uppsala University, Sweden

**Keywords:** photocatalysis, APXPS, photo-ALD, solar simulator

## Abstract

The Ambient-Pressure X-ray Photoelectron Spectroscopy (APXPS) endstation at the SPECIES beamline at MAX IV Laboratory has been improved. The latest upgrades help in performing photo-assisted experiments under *operando* conditions in the mbar pressure range using gas and vapour mixtures whilst also reducing beam damage to the sample caused by X-ray irradiation. This article reports on endstation upgrades for APXPS and examples of scientific cases of *in situ* photocatalysis, photoreduction and photo-assisted atomic layer deposition (photo-ALD).

## Abstract 2

A new experimental setup makes it possible to follow the evolution of surfaces *in situ* with ambient-pressure X-ray photoelectron spectroscopy by simultaneously irradiating the sample with ultra-violet and visible light. Photo-assisted processes can be carried out close to industrially relevant conditions.

## 1. Introduction

Processes driven by solar radiation play a key role in life on Earth. The first use of solar energy in chemical synthesis was made at the beginning of the 20th century by Giacomo Luigi Ciamician, who studied the behaviour of organic compounds under solar irradiation (Roth, 1989 ▸). Nowadays, replacing fossil fuels with clean and renewable energy sources is one of the most important challenges for modern human civilization (Dincer, 2000 ▸). Renewable energy sources are numerous with one of the most consistent and predictable being solar radiation. Indeed, natural photocatalytic reactions can be utilized to convert solar energy into usable chemical energy. When a photocatalyst is subjected to solar irradiation it generates electrons and holes. These photogenerated electrons and holes can be further utilized to harvest and store solar energy in the form of chemical energy with photocatalysis-driven reactions. Typical photocatalysts are metal oxides (TiO_2_, ZnO, WO_3_, MoO_3_, ZrO_2_, SnO_2_, α-Fe_2_O_3_ *etc.*) with a band gap of approximately 3 eV (Spasiano *et al.*, 2015 ▸). These photocatalysts can absorb light in the solar light spectrum or their band gap can be tuned to do so.

Another important application of solar photocatalysis is the purification of air and water. A wide variety of contaminants are released from industrially produced plants and agriculture into the water system increasing pollution in ground and surface water (Gomathi Devi & Krishnamurthy, 2011 ▸; Haque *et al.*, 2012 ▸). The removal of contaminants such as heavy metals, solvents, drugs, volatile chlorinated organic acids, pesticides, dyes, detergents and arsenic compounds is a crucial ecological challenge. Photochemical oxidation and photocatalytic degradation processes have been widely used for the environmental treatment of waste streams (Hupka *et al.*, 2006 ▸).

The sun plays a leading role in reactions and processes occurring in the atmosphere and in nature. In particular, the interaction between atmospheric particles and water under solar radiation is one of the most important processes in the Earth’s atmosphere. Solar light promotes reactions between SO_3_ and organic acid at the air–water interface, which are different from those in the gas phase and are important for the heterogeneous formation of H_2_SO_4_ and the subsequent formation of new particles (Lv & Sun, 2020 ▸; Zhong *et al.*, 2019 ▸). The composition of the droplet surface can affect mass transport and the chemical reactions that take place on the surface (Roy *et al.*, 2020 ▸).

Some industrial processes can be enhanced by photo-irradiation; one in particular is that of atomic layer deposition (ALD). ALD is a versatile thin-film deposition process that is currently used in many steps within the microelectronics process flow and is also gaining increased interest in other industrial sectors. Selective-area ALD is often attempted by passivating the non-film growth areas with self-assembled monolayers or polymers. However, it requires the removal of the passivation layer after deposition, or/and selectivity may be lost after a few ALD cycles. Photo-assisted ALD (photo-ALD) is a novel approach to facilitating selective-area ALD by simply masking areas without need for a passivation layer; however, the materials accessible through photo-ALD remain limited with poor film quality. Thus, there is great interest in *in situ* studies of photo-ALD (Henke *et al.*, 2015 ▸; Miikkulainen *et al.*, 2017 ▸, 2019 ▸). Our dedicated ALD cell at the SPECIES beamline fulfils this need by providing a platform to perform *in situ* ALD with easily exchangeable light sources for photo-ALD studies.

Today, ambient-pressure X-ray photoelectron spectroscopy (APXPS) is a widely used scientific technique that reveals chemical, electronic and mechanical properties of surfaces and interfaces on the nanometre scale. The APXPS branch and endstation at the SPECIES beamline on the 1.5 GeV storage ring at MAX IV Laboratory is designed to perform *in situ* and *operando* APXPS experiments in controlled atmospheres at pressures up to 20 mbar. A cell-in-cell X-ray photoelectron spectroscopy (XPS) endstation was developed at the former MAX-lab Laboratory (Knudsen *et al.*, 2016 ▸; Schnadt *et al.*, 2012 ▸) and installed at the SPECIES beamline at MAX IV Laboratory (Urpelainen *et al.*, 2017 ▸). The gas inlet system is done via a so-called double-cone system, where the inner cone has an aperture through which electrons enter the analyser and an outer, larger-diameter cone directs gas towards the sample surface from the same direction as the analyser (Kokkonen *et al.*, 2021 ▸). Recently, the dedicated ALD cell at SPECIES was designed and built to achieve gas flow that mimics the flow in real ALD reactors (Kokkonen *et al.*, 2022 ▸). APXPS has proven to be a very powerful tool for studying *in situ* and *operando* studies of ALD cycles and for revealing reaction steps otherwise unobservable with traditional methods (D’Acunto *et al.*, 2022 ▸; Temperton *et al.*, 2019 ▸; Timm *et al.*, 2018 ▸).

Main research areas accessible at the endstation include catalysis, material and surface sciences, environmental chemistry and ALD, to name a few. However, there is a lack of APXPS studies combining the technique with external light sources to study physicochemical changes with solid surfaces under conditions relevant for photochemical reactions. Here, we will introduce and discuss the use of UV and solar simulated light irradiance with APXPS cells at this particular instrument. We provide a series of scientific case studies from photocatalysis to photo-ALD that serve to illustrate the usefulness of the concept.

## 2. Improvement of the APXPS endstation

**Figure 1:**
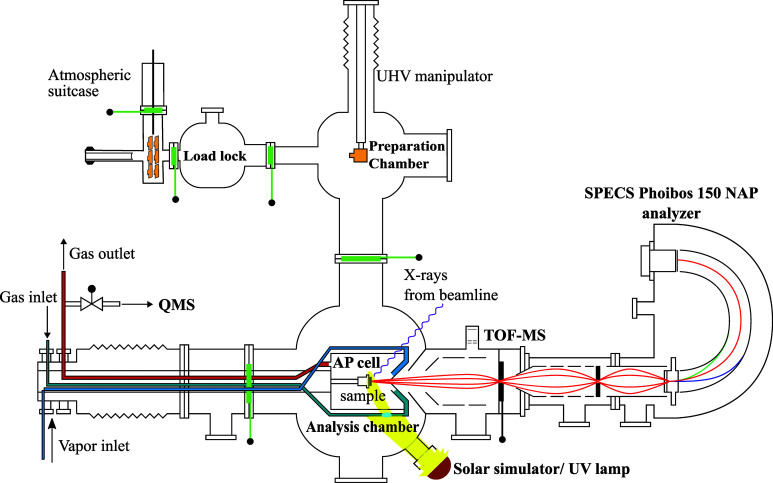
Schematic layout of the APXPS endstation at the SPECIES beamline at MAX IV Laboratory, consisting of a load lock chamber with a quick access door and optional atmospheric suitcase, preparation chamber with a UHV manipulator and option to attach user equipment, and analysis chamber housing an AP cell.

The earlier version of the APXPS setup at the SPECIES beamline at MAX IV Laboratory is described in detail in a previous publication (Kokkonen *et al.*, 2021 ▸). Briefly, the setup consists of three main chambers connected to a near-ambient-pressure analyser: load lock, preparation and analysis chambers with an exchangeable reaction (or AP) cell (Fig. 1 ▸). The load lock chamber has a quick-access door and a horizontal transfer to the preparation chamber. The preparation chamber is equipped with a sputter gun, low-energy-electron-diffraction system and port for user equipment. The endstation is equipped with a UHV manipulator to allow XPS and X-ray absorption spectroscopy (total and/or partial electron yield) normal measurements under UHV conditions with sample heating and some sample preparation methods. The analysis chamber has storage for three samples and an AP cell. Available cells include a standard cell for model and industrial catalysis and an ALD cell for ALD growth studies. The APXPS endstation is equipped with a PrismaPro 250 M2 quadrupole mass spectrometer (Pfeiffer) in the gas outlet line of the AP cell, a time-of-flight mass spectrometer (VACOM NOVION) in the pre-lens chamber to monitor gas phase contributions, and a SPECS Phoibos 150 NAP electron energy analyser.

**Figure 2:**
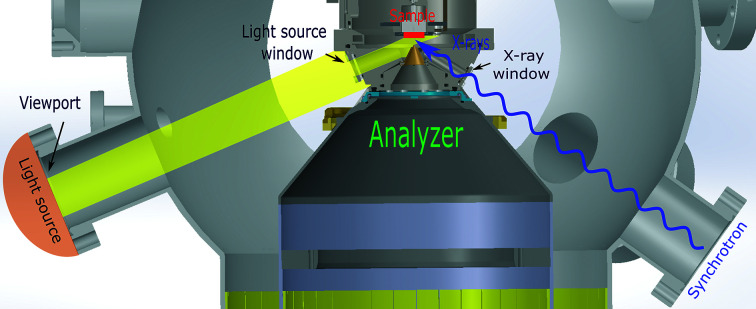
Schematic layout (top to bottom view) showing the alignment between external light sources, beamline and analyser nozzle in the analysis chamber with AP cell at the SPECIES beamline at MAX IV Laboratory.

The upgraded version of the endstation presented here has two external light sources including a HAL-320W solar simulator (Asahi Spectra) with a custom light guide and a pulsed UV lamp (Hamamatsu L6605) for the purpose of irradiating samples. Both light sources can be attached to a CF40 viewport with UV grade fused silica on the analysis chamber, allowing light to be used in either the UHV manipulator or the AP cell and at different endstations at the SPECIES and HIPPIE beamlines at MAX IV Laboratory. Fig. 2 ▸ schematically shows the alignment between external light sources, beamline and analyser nozzle. The angle between external light source and sample surface is 69°. At the same time, X-rays reach the sample at the ‘magic’ angle of 54.7°.

**Figure 3:**
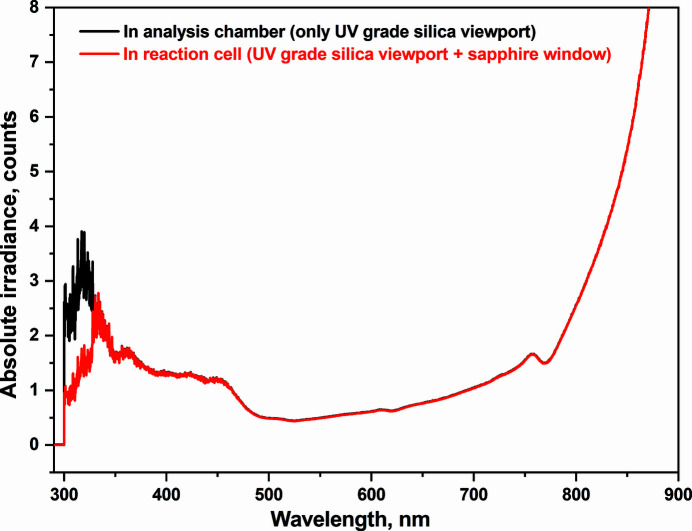
Spectrophotometer spectra measured in the analysis chamber (through a UV-grade fused viewport) (black line) and the reaction cell (through a UV-grade fused silica viewport and sapphire window) (red line) configuration.

Both the standard and ALD cells allow for *in situ* APXPS measurements of catalytic reactions and ALD growth processes, respectively. However, photocatalysis and photo-ALD require external light sources. Currently, two different light sources are available at the beamline: a solar simulator and a pulsed UV lamp. The HAL-320W solar simulator utilizes a 300 W short arc xenon lamp and generates light, which mimics the solar spectrum in the wavelength range between 350 nm and 1800 nm with a special Air Mass 1.5G filter. The circular variable neutral-density filter can be used to attenuate the light intensity. Typical sample sizes of 8 mm × 8 mm or 10 mm diameter at the SPECIES-APXPS setup are smaller than a uniform irradiation area of 18 mm × 18 mm at a working distance of approximately 224 mm. Fig. 3 ▸ shows spectrophotometric spectra of the solar simulator measured through a UV-grade fused silica viewport (black line) compared with a fused silica viewport and TECHSPEC sapphire window on the cell (Edmund Optics, red line), which mimics the analysis chamber and reaction cell geometries, respectively. The power density of the solar simulator was measured with a calibrated reference Si PV cell (Newport, model 91150V) and is approximately 0.5 sun (1 sun = 1000 W m^−2^) at the sample position inside the reaction cell.

Operation of either the solar simulator or the UV lamp requires good transmission into the vacuum chamber and subsequently the AP cell, thus special windows are necessary for specific light transmission. The analysis chamber can be equipped with standard borosilicate glass or UV-grade fused silica viewport. Currently, there are several options for cell windows on the reaction cell: (i) BOROFLOAT borosilicate windows (Edmund Optics) have a transmission of more than 90% between 350 and 2500 nm; (ii) TECHSPEC sapphire windows (Edmund Optics) are useful in the transmission range 330–5500 nm; (iii) fused quartz windows (Knight Optical) are a more suitable material choice for UV light; (iv) any other window materials of the correct size which satisfies the needs of the users’ experiment. User-supplied light sources can also be attached to the main chamber with minimal effort.

The solar simulator covers a wide range of light from visible (VIS) to infrared (IR). However, some experiments and applications require the use of UV light. Thus, an additional xenon flash lamp is required to extend into the UV range with single-crystal sapphire glass (Hamamatsu L6605) that generates light from 190 nm to 2000 nm. The pulsed lamp has a variable repetition rate of 10–100 Hz and variable intensity leading to tunable lamp power.

Additionally, the load lock chamber of the endstation has been improved with the addition of an atmospheric suitcase. The atmospheric suitcase has six slots for SPECS/Omicron-style sample plates and allows sample transfer to the experimental station without exposure to air. In fact, air-sensitive samples can be transferred directly from a glove box or another setup to the load lock or vice versa.

A rotatable beam shutter was also installed between the exit slit and the M4 refocusing mirror of the APXPS beamline branch to address the common problem of beam damage (due to high photon density of undulator beamlines) on X-ray sensitive samples. The actuation of the shutter can be automatically triggered by the voltage on the detector of the electron analyser when acquiring a spectrum. Therefore, the sample is only exposed to X-rays when spectra are recorded. This logic minimizes unnecessary X-ray exposure of the sample in between data acquisitions and acts to reduce the degradation of samples under X-ray flux. Furthermore, beam damage can be avoided by raster scanning the sample during the measurements.

The sample heating system in the standard cell has also been improved. The previous system was based on resistive heating with a button heater (a platinum filament enclosed in Al_2_O_3_). However, the platinum wire on the heater turned out to be catalytically active for numerous reactions studied in the standard cell leading to contaminated mass spectrometer data. Moreover, the spot weld between the platinum wire and the molybdenum feedthrough of the heater was fragile which led to many issues. The upgraded version of the sample heating system addresses both of these issues. It consists of a pyrolytic graphene (PG) track inside a pyrolytic boron nitride (PBN) insulator with a 9 mm-diameter heating surface (model PCPBNS01, Thermic Edge). As specified, the PG-PBN is chemically inert to corrosive gases, liquids and most molten metals and is suitable for use in ultra-high vacuum up to 1600°C and in air up to 500°C.

## 3. Results and discussion of scientific cases

To test the improved setup and verify its performance, several experimental cases were selected: two examples from photocatalysis and one from photo-assisted ALD. In this section we present these examples from the point of view of the technical feasibility of the system and the potential it offers. More detailed discussions and expanded datasets on the cases presented are left for later publications, which focus on the scientific results of the experiments in detail.

### 3.1. Ni@NiO/NiCO_3_ core–shell nanostructures for photocatalytic hydrogen evolution

Over the past few decades, renewable energy has received researchers’ attention due to the limited supply of fossil fuels and their negative impact on the environment such as escalating pollution and global warming (Babayan *et al.*, 2019 ▸; Panwar *et al.*, 2011 ▸). Green hydrogen is the most sustainable substitute for fossil fuels; however, the production of cost-effective green hydrogen is still a challenge. In this regard, photocatalytic water splitting is considered one of the more promising ways to generate environmentally friendly and inexpensive hydrogen. However, even after intensive research efforts put into photocatalytic hydrogen production around the world, this energy source remains elusive due to the more prominent and less efficient hydrogen evolution reaction (HER), necessitating continued work in this area. In a recent study by Talebi *et al.* (2021 ▸), pristine Ni@NiO/NiCO_3_ core–shell nanostructures and their post-annealing variants have been characterized and evaluated for photocatalytic HER. A thorough structural (X-ray diffraction) and spectroscopic (XPS, transmission electron microscopy/electron energy-loss spectroscopy) investigation revealed that the core of the Ni nanoparticle is decorated with a shell (∼3–5 nm thick) composed of a composite of crystalline NiO and amorphous NiCO_3_. The nanoparticle has significant visible light absorption at ∼475 nm suggesting that the surface plasmon resonance excitation plays a role in the observed catalytic activity. Furthermore, it was found that the amount of NiCO_3_ in the shell positively correlates with HER activity. However, the exact role of NiCO_3_ is still unknown (Patra *et al.*, 2020 ▸; Zilberg *et al.*, 2018 ▸). To test the feasibility of the upgraded setup for photocatalytic HER, we have studied the reaction mechanisms of HER on Ni@NiO/NiCO_3_ core–shell nanocatalysts monitoring electronic structure evolution using APXPS under solar simulator irradiance through a fused silica viewport and borosilicate window.

**Figure 4:**
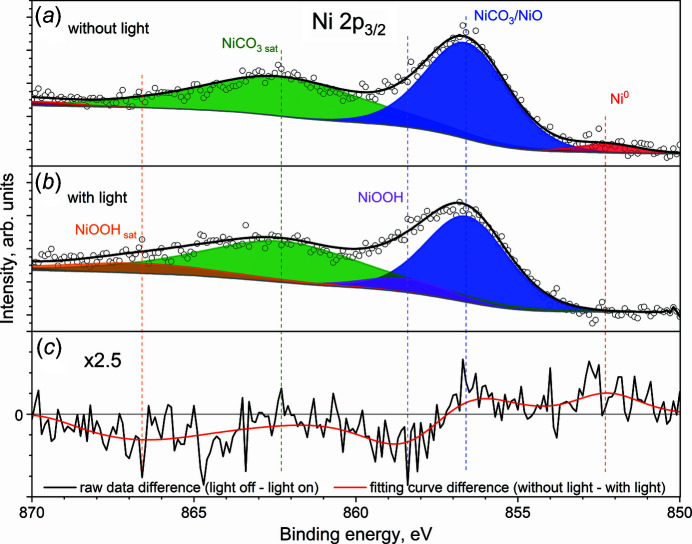
Ni 2*p*_3/2_ spectrum of *in situ* APXPS studies of HER on Ni@NiO/NiCO_3_ at room temperature under 1 mbar H_2_O (*a*) without and (*b*) with simulated light through a fused silica viewport and borosilicate window, and (*c*) the difference spectrum.

The nanocatalyst sample was drop-casted onto Au foil. The *in situ* spectroscopic experiments were performed under 1 mbar of water vapour at room temperature with solar-simulated light illumination cycling between an on and off state. The evolution of Ni 2*p*, C 1*s* and O 1*s* core-levels was monitored. First, 1 mbar of water vapour was dosed into the reaction cell at room temperature and XPS spectra were recorded with and without light irradiation. As shown in Fig. 4 ▸, the Ni 2*p* spectrum has a complex shape resulting from multiplet splitting, shake-up and plasmon loss structures (Biesinger *et al.*, 2009 ▸, 2011 ▸). Without visible light illumination on the catalyst surface, the Ni 2*p*_3/2_ structure [Fig. 4 ▸(*a*)] can be fitted well with three components corresponding to different chemical states of Ni (Ni^0^, NiO and NiCO_3_). The small peak (red) at binding energy (BE) of 852.1 eV can be assigned to metallic Ni (Biesinger *et al.*, 2009 ▸, 2011 ▸). The most intense peak (blue) at BE of 856.5 eV and a satellite (green) at BE of 862.1 eV belong to Ni^2+^ (Heine *et al.*, 2016 ▸; López-Rodríguez *et al.*, 2022 ▸) (Table S1 of the supporting information).

During light illumination [Fig. 4 ▸(*b*)], it was observed that the metallic Ni peak vanishes and a new peak (magenta) with a satellite (orange) appears at BE of 858.4 eV and 866.5 eV, respectively. However, no changes were observed in the Ni 2*p* peaks from NiO and/or NiCO_3_. We speculate that a newly developed peak at BE of 857.8 eV can most likely be assigned to NiOOH (Biesinger *et al.*, 2011 ▸, 2012 ▸).

Indeed, the difference spectrum [Fig. 4 ▸(*c*)] shows positive values in the region below BE = 567 eV, indicating a higher contribution from Ni^0^ and NiCO_3_/NiO in the absence of light. At the same time, negative values in the high BE range represent development of new features of NiOOH only with light illumination.

These APXPS studies show that the metallic nickel absorbs the light and is oxidized to NiOOH in the presence of H_2_O vapour. The NiOOH species are only present under light illumination conditions. However, when the light illumination was switched off, NiOOH was reduced back to metallic nickel. The results were reproducible over multiple cycles of light exposure.

### 3.2. Pd–TiO_2_ catalysts

TiO_2_-based photocatalysts have been used in many applications and are the most widely studied due to their chemical stability, low cost, non-toxicity and strong oxidizing ability, especially for air and water purification systems (Nakata & Fujishima, 2012 ▸). Metal nanoparticles such as silver, gold and palladium have a broad function and the ability to capture electrons generated by light to inhibit recombination (Kochuveedu *et al.*, 2013 ▸; Melvin *et al.*, 2015 ▸; Patra & Gopinath, 2016 ▸; Wu *et al.*, 2013 ▸; Zhao *et al.*, 2012 ▸). Here, we investigate the stability of a Pd–TiO_2_ surface under solar irradiation through a fused silica viewport and borosilicate window. In this work, Pd–TiO_2_ catalyst was synthesized by following the procedure reported in the literature (Nalajala *et al.*, 2019 ▸).

**Figure 5:**
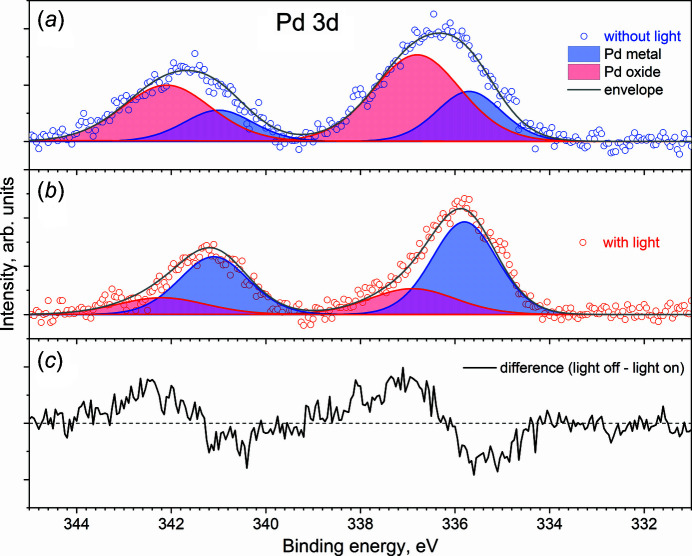
Pd 3*d* XPS spectra of Pd–TiO_2_ catalyst in UHV (*a*) without and (*b*) with light illumination through a fused silica viewport and borosilicate window and (*c*) the difference spectrum.

Fig. 5 ▸(*a*) shows the Pd 3*d* XP spectrum in UHV without solar irradiation. The spectrum consists of two broad peaks with full width at half-maximum (FWHM) of 1.84 eV and 2.15 eV at BE of 336.3 eV and 341.6 eV, corresponding to the spin–orbit splitting of Pd 3*d*_5/2_ and Pd 3*d*_3/2_, respectively. After turning on the solar simulator [Fig. 5 ▸(*b*)], the Pd 3*d* peaks narrow and have FWHM of 1.40 eV and 1.73 eV, respectively. Fig. 5 ▸(*c*) shows the difference spectrum of Pd 3*d* with and without light illumination. High BE components at BE of 337.0 eV and 342.2 eV disappear significantly under solar radiation (Table S3). At the same time, the intensity of the components at BE of 335.5 eV and 342.1 eV increases. According to the literature, the low BE features can be attributed to metallic Pd (Bukhtiyarov *et al.*, 2018 ▸; Panafidin *et al.*, 2019 ▸; Venezia *et al.*, 2003 ▸), and high BE peaks, shifted by 1.5 eV from the position of metallic Pd, are usually assigned to palladium oxide (Peuckert, 1985 ▸; Jeong *et al.*, 2021 ▸). The TiO_2_ support remains unchanged throughout all experiments (Fig. S3 of the supporting information). This underscores the photoreduction of palladium oxide taking place under solar radiation. This observation directly indicates the presence of abundant metal–semiconductor (Schottky) junctions between Pd and TiO_2_ (Dubey *et al.*, 2022 ▸; Nalajala *et al.*, 2019 ▸), which contributes to the separation of electron–hole pairs at the junction and electrons are stored in Pd. We also observed an irreversible photoreduction of Pd (Fig. S2). Blank experiments were performed with only X-rays to exclude the impact of the beam damage. No significant change of Pd 3*d* was observed under X-ray exposure for the duration of our studies within 2 h (Fig. S3).

### 3.3. Photo-assisted ALD

Another example lies in an under-utilized technique called photo-ALD which allows for spatially selective ALD to be carried out. A photo-assisted ALD deposition sequence consists of a precursor pulse, a purge, light exposure, and another purge as opposed to traditional ALD deposition that uses two precursors and no light irradiation. Thus, the incorporation of light irradiation replaces the need for two precursors and instead promotes the reaction via photocatalysis. For example, photo-ALD of metal oxides from alkoxides as a single precursor has been an established technique, and it has demonstrated selective ALD by shadow masking (Miikkulainen *et al.*, 2019 ▸, 2021 ▸). The reaction mechanism is most likely similar to those reported by Anderson *et al.* for titanium tetraisopropoxide [Ti(OiPr)_4_] in a more conventional ALD process: an elimination of the adsorbed isopropoxide releases propene leaving –OH on the surface to react further (Anderson *et al.*, 2013 ▸). In photo-ALD, a photoinduced step most likely initiates the elimination. The OH is reactive towards the following Ti(OiPr)_4_ pulse, which forms a TiO_2_ film. On the other hand, unreacted Ti(OiPr)_4_ does not adsorb on the shaded areas of the film, thus stunting TiO_2_ production under the masked regions. Recently, Miikkulainen *et al.* reported on a detailed characterization of the deposition of five metal oxides on Si(100) from alkoxide precursors of particular interest, namely Nb_2_O_5_, Ta_2_O_5_, TiO_2_, HfO_2_ and ZrO_2_, which have been shown to deposit as either amorphous or crystalline films depending on the deposition conditions (Miikkulainen *et al.*, 2019 ▸). Interpreting the evolution of these responses in relation to UV exposure is critical to assessing the photo-ALD process alongside confirming the role of the substrate for a better understanding of the entire photo-ALD process. These studies promote the discovery of new photo-ALD processes and substrate/photo-ALD precursor pairs.

**Figure 6:**
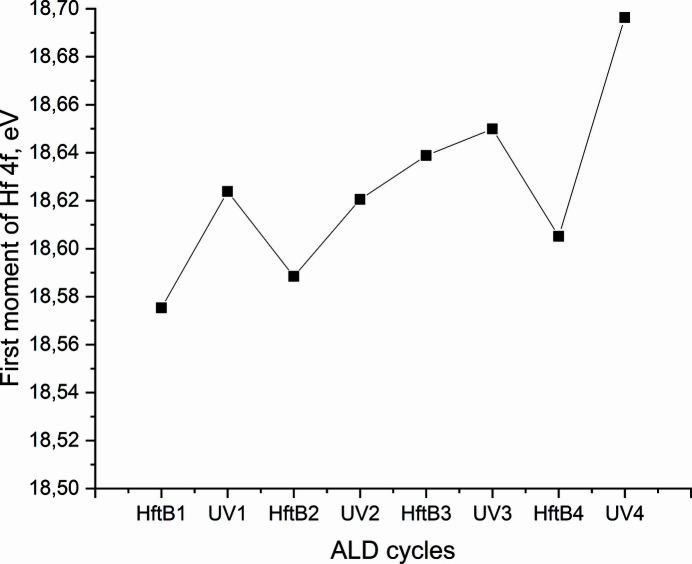
The change of the first moment of the Hf 4*f* core level over four cycles of a hafnium *tert*-butoxide [Hf(OtBu)_4_] precursor and UV pulses through a sapphire viewport and sapphire window.

Herein, we tested the photo-ALD setup using a hafnium *tert*-butoxide [Hf(OtBu)_4_] precursor and UV light to produce HfO_2_ on an Si(100) wafer. An Xe lamp (190–2000 nm) was mounted on a UV-grade fused silica viewport on the analysis chamber to provide the UV pulses through a sapphire window. Throughout the deposition, XPS was recorded with time-resolved snapshot mode tracking a series of pertinent core-levels every 15 s. Fig. 6 ▸ shows the first moment of the Hf 4*f* peak (Fig. S6, Table S4) over four cycles as measured with time-resolved XPS. The Hf 4*f*_7/2_ BE for the oxide is between 16.4 and 19.1 eV (Cho *et al.*, 2002 ▸; Chourasia *et al.*, 2009 ▸; Fang *et al.*, 2004 ▸; Hernández-Arriaga *et al.*, 2017 ▸; Kaichev *et al.*, 2011 ▸; Renault *et al.*, 2002 ▸; Sha *et al.*, 2003 ▸; Smirnova *et al.*, 2008 ▸; Ohtsu *et al.*, 2007 ▸). The first moment of the Hf 4*f* peak moves towards a higher binding energy during UV exposure suggesting that HfO_*x*_ is being formed during the UV pulse.

## 4. Conclusions

The APXPS endstation at the SPECIES beamline at MAX IV Laboratory has a standard ambient-pressure cell for catalysis, redox chemistry, *etc*., and an ALD ambient-pressure cell for ALD growth studies. Two external light sources (solar simulator or UV lamp) can be used with either AP cell. Coupling the light source to the ambient-pressure cell provides a unique platform for experiments studying the electronic structure of matter under infrared, visible or UV radiation at relevant conditions near ambient pressure. In this article, we demonstrate APXPS analysis of photoinduced processes that provide information to advance and improve our current knowledge of different reaction mechanisms in nature and industry that are possible at the SPECIES beamline of MAX IV Laboratory. The solar simulator and UV lamp are currently available for users at both SPECIES and HIPPIE beamlines (Zhu *et al.*, 2021 ▸) at MAX IV Laboratory with a similar ambient-pressure cell for APXPS that can perform simultaneous infrared reflection–absorption spectroscopy (IRRAS).

## Supplementary Material

Supporting Figures S1 to S7, Tables S1 to S4. DOI: 10.1107/S1600577523002801/ve5168sup1.pdf
