# Supplementary material for: Photocatalytic setup for in situ and operando ambient-pressure X-ray photoelectron spectroscopy at MAX IV Laboratory
Source: J Synchrotron Radiat. 2023 Apr 17;30(Pt 3):613–9. doi: 10.1107/S1600577523002801 (PMC10161884; doi:10.1107/S1600577523002801)
Supplement: Supplementary file 1 [file s-30-00613-sup1.pdf]

JOURNAL OF SYNCHROTRON RADIATION

Volume 30 (2023)

Supporting information for article:

Photocatalytic set-up for in situ and operando ambient pressure X-ray photoelectron spectroscopy at the MAX IV laboratory

Alexander Klyushin, Manoj Ghosalya, Esko Kokkonen, Calley Eads, Rosemary Jones, Naresh Nalajala, Chinnakonda S. Gopinath and Samuli Urpelainen

For quantitative XPS analysis least-squares fitting of Ni2p spectra was made using CasaXPS software (www.casaxps.com). A Gaussian/Lorentzian product was used to obtain the best fit. The binding energy of the components was constrained to be the same in all spectra and conditions. All fitting parameters are given in the Table S1 (multiple peaks and satellites) and Table S2 (one peak and satellite). Residual spectra (Figures S1c-d and S2c-d) show a better fit with multiple peaks and satellites.

Table S1 Fit parameters of Ni $2p_{3/2}$ XP spectra of Ni@NiO/$NiCO_3$ (multiple components and satelites)

	Ni^0	NiO/$NiCO_3$	NiOOH	NiO/$NiCO_{3\ sat}$	$NiOOH_{sat}$
Conditions			Without light		
Lineshape	GL	GL	-	GL	-
Binding energy, eV	852.2	856.6	-	862.2	-
FWHM, eV	2.3	3.0	-	6.0	-
			With light		
Lineshape	-	GL	GL	GL	GL
Binding energy, eV	-	856.6	858.4	862.3	866.6
FWHM, eV	-	3.0	3.0	6.0	6.0

GL - Gaussian–Lorentzian; Ni 2p spin-orbital splitting of 17.5 eV.

Table S2 Fit parameters of Ni $2p_{3/2}$ XP spectra of Ni@NiO/$NiCO_3$ (one component and satelite)

	1st peak	satelite
Conditions	Without light	
Lineshape	GL	GL
Binding energy, eV	856.5	861.8
FWHM, eV	2.9	8.7
	With light	
Lineshape	GL	GL
Binding energy, eV	856.5	861.8
FWHM, eV	2.9	8.7

GL - Gaussian–Lorentzian; Ni 2p spin-orbital splitting of 17.5 eV.

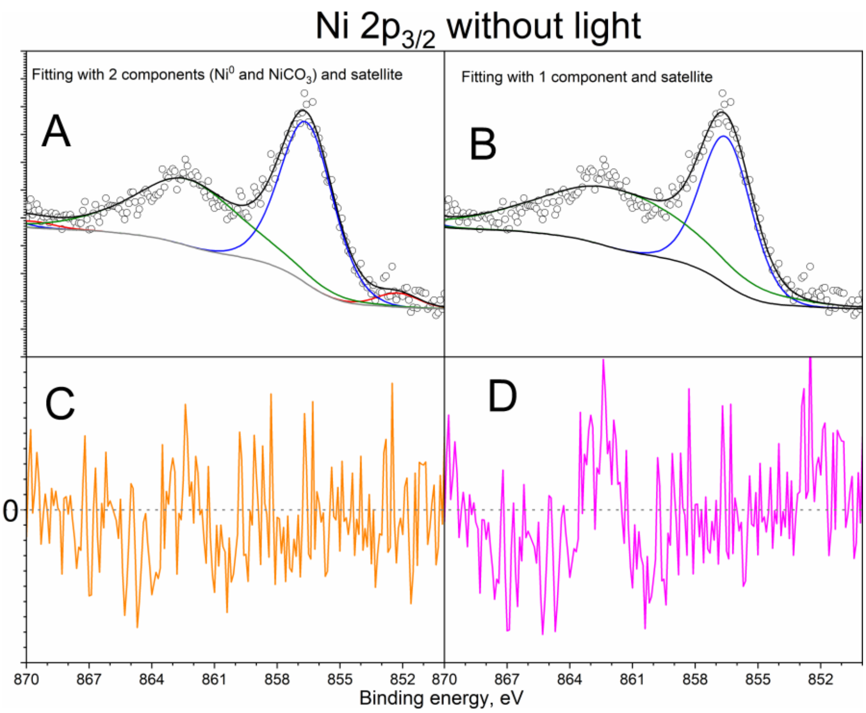

Figure S1 Fitting of Ni 2p3/2 XP spectra witthout of Ni@NiO/$NiCO_3$ catalyst with (A) 2 components ($NiCO_3$ and Ni^0) and satelite and (C) corresponding residuum, (B) 1 component and satellite and (D) corresponding residuum without light irradiation.

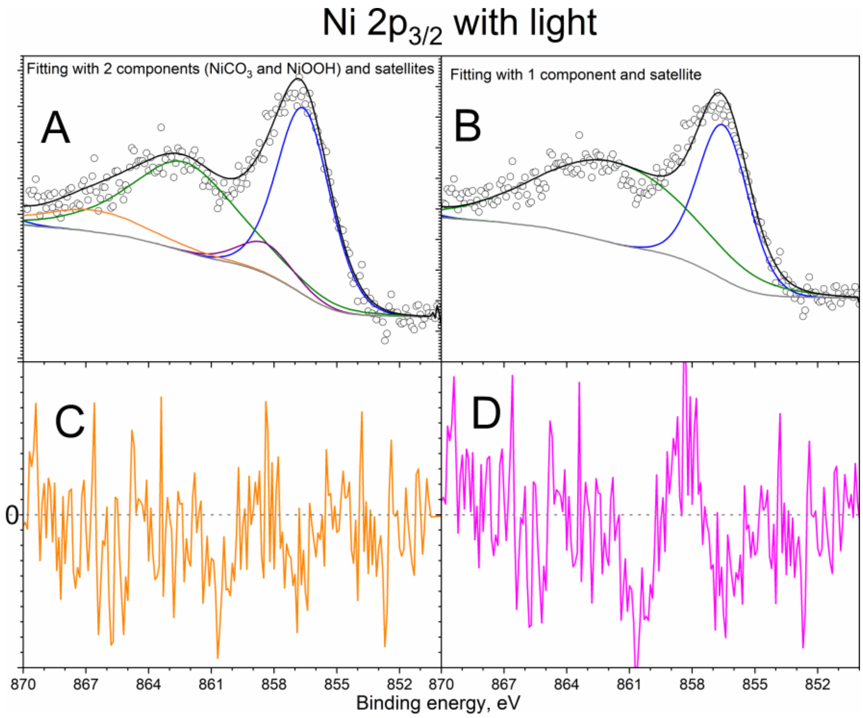

Figure S2 Fitting of Ni 2p3/2 XP spectra witthout of Ni@NiO/$NiCO_3$ catalyst with (A) 2 components ($NiCO_3$ and NiOOH) and satelites and (C) corresponding residuum, (B) 1 component and satellite and (D) corresponding residuum with light irradiation.

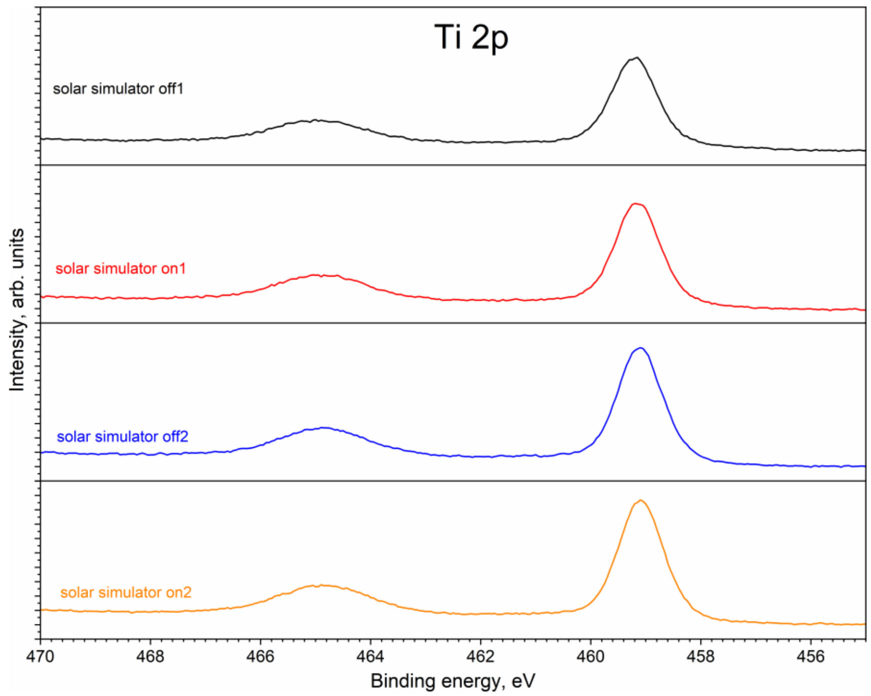

Figure S3 Ti 2p XP spectra of Pd-TiO_2 catalyst during two cycles of light irradiation.

Table S3 Fit parameters of Pd $3d_{5/2}$ XP spectra of Pd-TiO_2

	Pd metal	Pd oxide	Pd metal	Pd oxide
Conditions	Without light		With light	
Lineshape	GL	GL	GL	GL
Binding energy, eV	335.7	336.8	335.8	336.9
FWHM, eV	1.6	2.1	1.6	2.1

GL - Gaussian–Lorentzian; Pd 3d spin-orbital splitting of 5.3 eV.

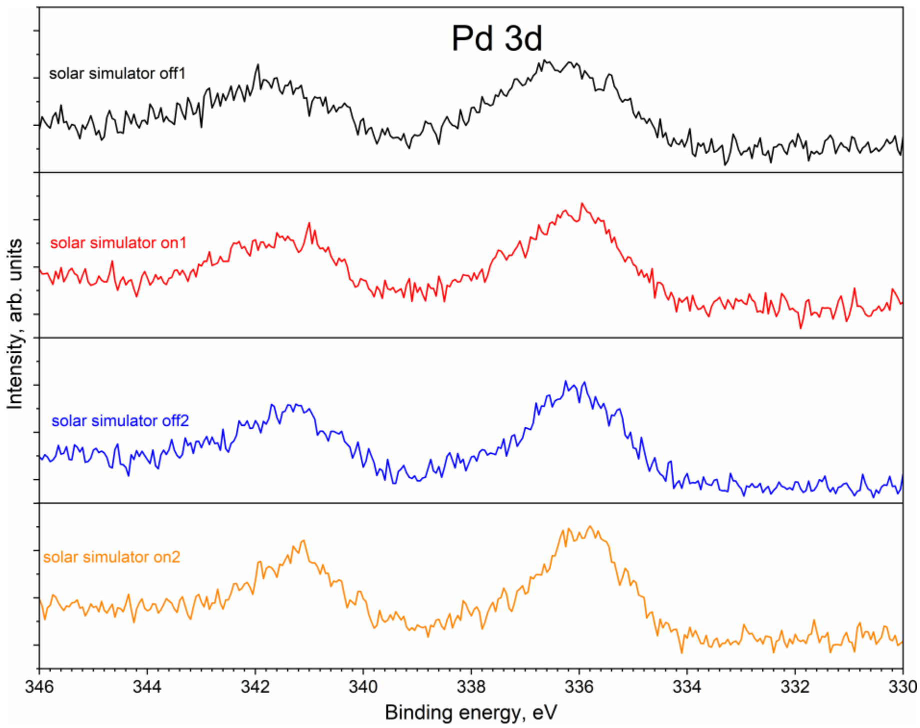

Figure S4 Pd 3d XP spectra of Pd-TiO_2 catalyst during two cycles of light irradiation.

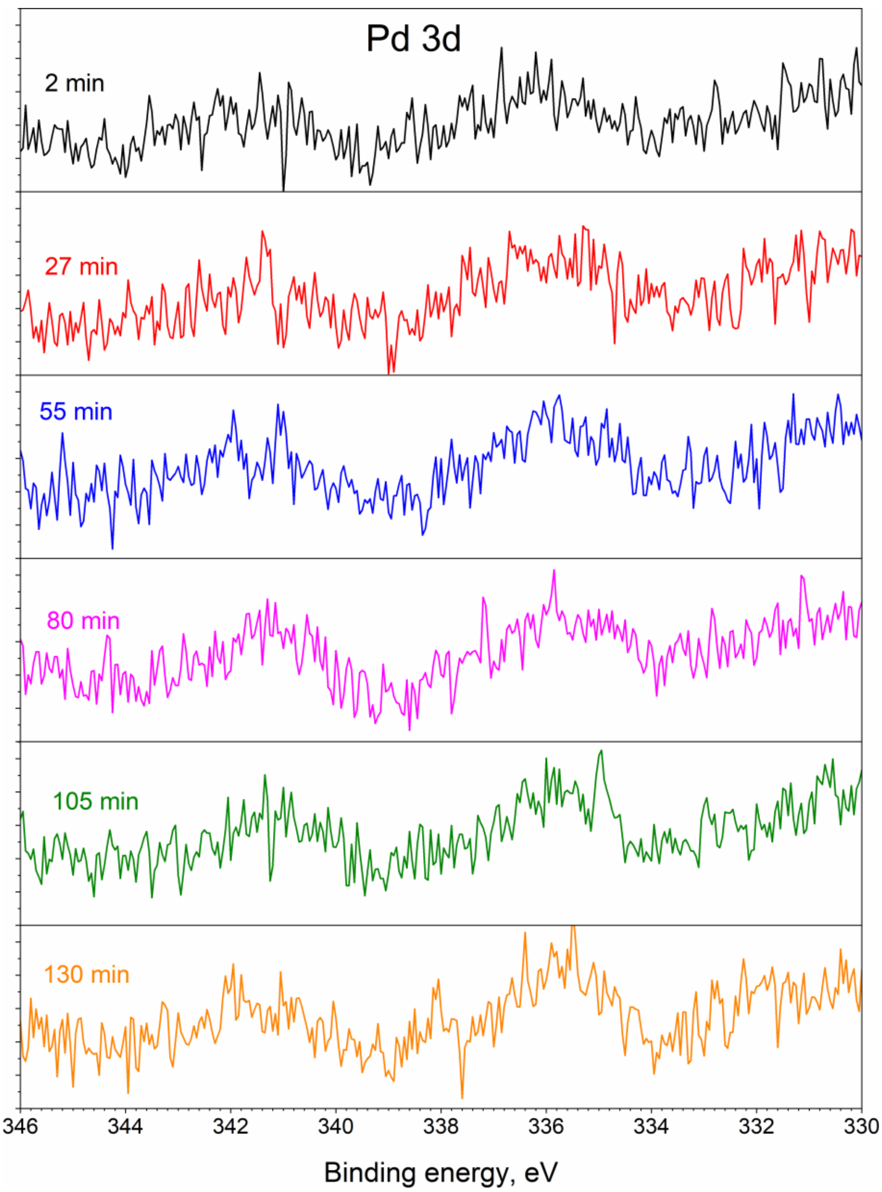

Figure S5 Pd 3d XP spectra of Pd-TiO_2 catalyst under X-ray flux for 2 hours.

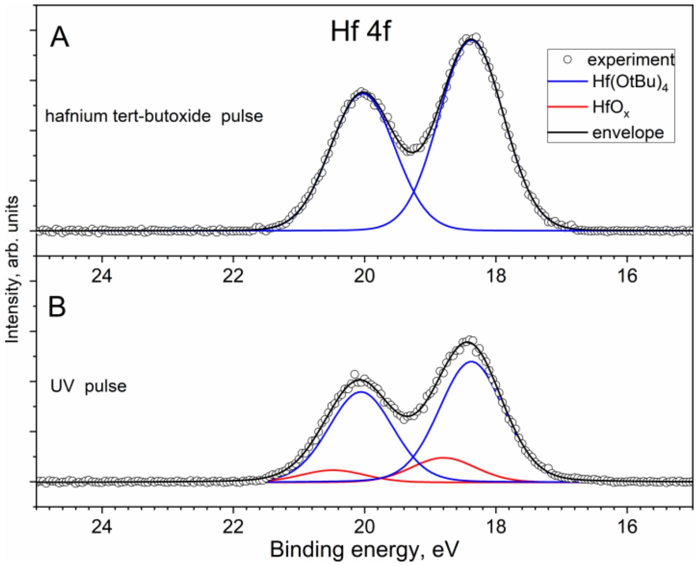

Figure S6 Hf 4f XP spectra over 1st cycle of (A) a hafnium tert-butoxide ($Hf(OtBu)_4$) and (B) UV pulses through a UV grade fused silica viewport and sapphire window.

Table S4 Fit parameters of Hf $4f_{7/2}$ XP spectra over 1st cycle of a hafnium tert-butoxide ($Hf(OtBu)_4$) and UV pulses.

	$Hf(OtBu)_4$	HfO_x	$Hf(OtBu)_4$	HfO_x
Conditions	Without light		With light	
Lineshape	GL	-	GL	GL
Binding energy, eV	18.3	-	18.3	18.8
FWHM, eV	1.1	-	1.1	1.1

GL - Gaussian–Lorentzian; Hf 4f spin-orbital splitting of 1.7 eV.

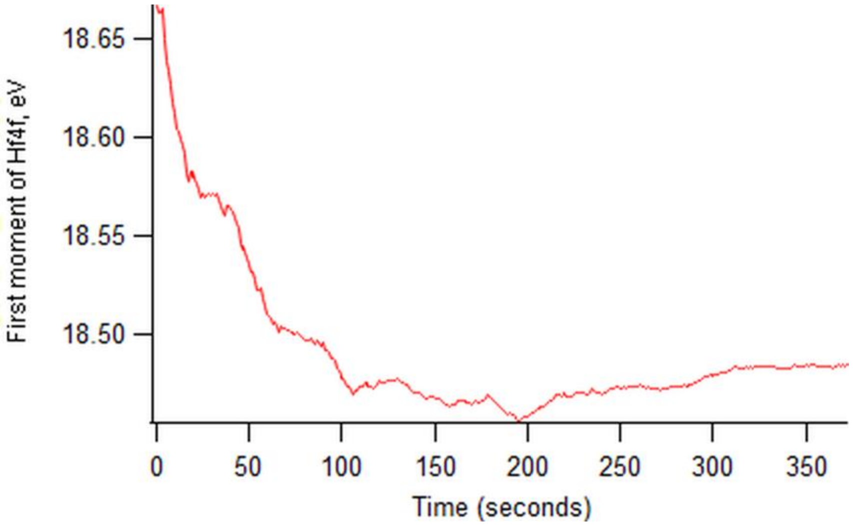

Figure S7 The first moment of the Hf 4f with time of exposures of a hafnium tert-butoxide (Hf(OtBu)4).
